# High BMI is associated with lower TNF-α inhibitor serum trough levels and higher disease activity in patients with axial spondyloarthritis

**DOI:** 10.1186/s13075-023-03187-4

**Published:** 2023-10-17

**Authors:** Liseth de Wolff, Suzanne Arends, Elisabeth Brouwer, Hendrika Bootsma, Anneke Spoorenberg

**Affiliations:** Department of Rheumatology and Clinical Immunology, University of Groningen, University Medical Center Groningen, Groningen, the Netherlands

**Keywords:** Axial spondyloarthritis, TNF-α inhibitors, BMI, Serum trough levels, Disease activity

## Abstract

**Background:**

TNF-α inhibitor (TNFi) serum trough levels have previously been found to be related to disease activity in axial spondyloarthritis (axSpA). However, most research regarding serum trough levels has been conducted in patients who only recently started TNFi therapy. Therefore, our objective was to explore TNFi serum trough level measurements in relation to disease activity and BMI in the total axSpA population in daily clinical practice, also including patients on long-term TNFi therapy.

**Methods:**

Consecutive patients from the Groningen Leeuwarden Axial Spondyloarthritis (GLAS) cohort were approached for a TNFi serum trough level measurement during their regular outpatient visit at the UMCG. Spearman’s correlation coefficient was used to analyse the relation of serum trough levels with disease activity and BMI. Logistic regression was performed to analyse the relation between therapeutic drug levels and disease activity, corrected for potential confounders, including BMI.

**Results:**

Thirty-four patients on adalimumab and 21 patients on etanercept were included. Mean age was 45 ± 12 years, 47% were male, median BMI was 26.4 (IQR 23.9–32.5) and median treatment duration was 41 months (range 2–126). According to definitions of Sanquin, 47% of patients had therapeutic serum trough levels. No significant correlations were found between TNFi levels and disease activity (ASDAS-CRP: adalimumab: ρ = -0.16, *p* = 0.39; etanercept: ρ = -0.29, *p* = 0.20). TNFi levels were moderately correlated with BMI (adalimumab: ρ = -0.48, *p* = 0.004; etanercept: ρ = -0.50, *p* = 0.021). Patients with active disease (ASDAS ≥ 2.1) showed higher BMI than patients with inactive disease (median 29.7 vs. 24.6, *p* = 0.015). In multivariable regression analyses, BMI was identified as the only confounder for the relationship between therapeutic drug levels and ASDAS.

**Conclusion:**

In this cross-sectional, observational study of axSpA patients mainly on long-term treatment with TNFi, higher BMI was significantly associated with lower adalimumab and etanercept serum trough levels and higher disease activity.

**Supplementary Information:**

The online version contains supplementary material available at 10.1186/s13075-023-03187-4.

## Introduction

Axial spondyloarthritis (axSpA) is a chronic, inflammatory rheumatic disease which mainly affects the spine and sacroiliac joints. The treatment of axSpA usually consists of non-steroidal anti-inflammatory drugs (NSAIDs) and physical therapy. If this first treatment fails to control disease activity, the next step is to start with biological treatment, e.g. tumour necrosis factor alpha inhibitor (TNFi) therapy [1]. However, TNFi treatment may fail, and is discontinued in approximately half of the patients, which can be related to persistently high disease activity, loss of treatment response or side effects [2, 3]. Previous studies have shown that lower TNFi serum trough levels are associated with an impaired initial therapeutic response in axSpA patients [4, 5]. Presence of anti-drug antibodies has been found to be one of the reasons for low drug levels and high disease activity [6, 7]. Furthermore, high body mass index (BMI) has been suggested as another explanation for low drug levels as some studies in axSpA patients found associations of high BMI with an impaired response to TNFi and lower TNFi serum trough levels [8, 9].

Most of the studies on therapeutic response and serum trough levels primarily involved patients on short-term treatment, often within 1 year [4, 7]. Only few studies have examined patients undergoing treatment for a longer duration. One study in 94 SpA patients showed an association between serum trough levels of infliximab and levels of disease activity, as measured with Ankylosing Spondylitis Disease Activity Score (ASDAS), at 6 months, 1 year and 4 years of treatment [6]. On the other hand, a retrospective study involving 81 SpA patients treated with adalimumab, etanercept or infliximab for a median treatment duration of 6, 30 and 54 months respectively, found no significant associations of serum trough levels with therapeutic response, defined as ASDAS < 2.1 [10].

Additionally, studies have been performed to determine cut-off values for therapeutic TNFi serum levels with mixed results. A study in 221 patients with rheumatoid arthritis (RA) showed that the optimal cut-off point for adalimumab trough levels was 5 μg/ml, based on the distinction between good responders and non or moderate responders based on the European Alliance of Associations for Rheumatology (EULAR) response criteria, showing a sensitivity of 91% and specificity of 43% [11]. A similar study in 102 AS patients was not able to show an association between adalimumab trough levels and therapeutic response, when evaluating change in disease activity (∆ASDAS and ∆Bath Ankylosing Spondylitis Disease Activity Index (BASDAI)) [12]. In a separate study of 292 RA patients on etanercept, it was observed that 40% of non-responding patients had etanercept trough levels below 2.1 mg/l. This study suggested that it might be useful to assess the effect of dosage increase in such cases [13]. Similarly, a study in 162 AS patients receiving etanercept yielded comparable results: 35% of patients with ongoing active disease according to ASDAS (≥ 2.1) exhibited low etanercept trough levels of < 1.8 mg/l. This study could not identify a clear therapeutic drug level window for etanercept [5]. Sanquin, a Dutch national laboratory for diagnostic services, defined a below therapeutic adalimumab trough level as < 5 μg/ml and, until 2019, a below therapeutic etanercept trough level as < 2 μg/ml [14].

Since most research regarding serum trough levels has been conducted in patients who only recently started TNFi therapy, our objective was to explore TNFi serum trough level measurements in relation to disease activity and BMI in the total axSpA population in daily clinical practice, also including patients on long-term TNFi therapy.

## Methods

### Groningen Leeuwarden Axial Spondyloarthritis (GLAS) cohort

The Groningen Leeuwarden Axial Spondyloarthritis (GLAS) cohort is an ongoing prospective longitudinal observational cohort study [15]. This cohort includes axSpA patients, fulfilling the modified New York (NY) criteria and/or the Assessment of SpondyloArthritis international Society (ASAS) classification criteria [15–17]. The local ethics committees of the Medical Centre Leeuwarden (MCL) and University Medical Centre Groningen (UMCG) approved the GLAS cohort, and all patients provided written informed consent according to the Declaration of Helsinki.

### Study patients

All consecutive patients from the UMCG on TNFi with a regular outpatient GLAS visit between June 2015 and June 2016 were asked to perform a single TNFi serum trough level measurement. This measurement was not performed because of a clinical indication, such as an increase in disease activity. Clinical data from GLAS visits closest to the serum trough level measurements were used. TNFi serum trough level was defined as a TNFi serum level measurement shortly before (on the same day or the day prior) the next drug administration. Patients were excluded from the analyses if their serum TNFi level did not fulfil the definition of a trough level measurement and/or if clinical data collection exceeded a period of more than two months from the serum trough level measurement date.

### Treatment with TNFi

Treatment with TNFi was initiated because of active disease according to the ASAS consensus statement [18]. The selection of the specific TNFi was determined by the rheumatologist’s clinical judgement and/or the specific preference of the patients. The following TNFi were used: infliximab, etanercept, adalimumab, golimumab and certolizumab. The standard regimens of the different TNFi were: for adalimumab (40 mg) subcutaneous injections once per two weeks; for etanercept (50 mg) subcutaneous injections once a week; for infliximab 5 mg/kg intravenously at 0, 2 and 6 weeks when starting treatment and then every 8 weeks; for golimumab (50 mg) subcutaneous injections once a month; for certolizumab (200 mg) subcutaneous injections once per two weeks. The dosing regimen could be adjusted in response to disease activity, side effects or co-morbidity. Patients were allowed to receive concomitant medication as usual.

### TNFi serum trough levels and cut-off values

The methods of measurement of TNFi trough serum levels have been described previously [19]. In short, TNFi trough serum levels were measured by enzyme-linked immunosorbent assay (ELISA: Sanquin, Amsterdam [20]). Based on reference values of Sanquin, serum trough levels were split into a therapeutic and below therapeutic range [14]. Sanquin published until 2019 the following definitions of below therapeutic cut-off trough levels for TNFi: adalimumab < 5 µg/ml, etanercept < 2 µg/ml and infliximab < 1 µg/ml. For golimumab and certolizumab, there are no clear therapeutic cut-off levels. If serum trough levels were in the below therapeutic range, anti-drug antibodies were assessed. For etanercept, anti-drug antibodies cannot be measured. High avidity IgG anti-drug antibodies to TNFi were detected by radioimmunoassay (RIA: Sanquin, Amsterdam) [14].

### Clinical data including disease activity

Clinical data were collected during the GLAS visit according to the GLAS protocol [15] and included patient characteristics including, among other characteristics, BMI and the following disease activity assessments: ASDAS, BASDAI and C-reactive protein (CRP). Cut-off values for active disease were ≥ 2.1 for ASDAS, ≥ 4.0 for BASDAI and ≥ 5.0 mg/L for CRP [18, 21].

### Statistical analyses

The statistical analysis was conducted using Statistical Package for the Social Sciences (SPSS) version 23.0. Group differences in patient characteristics and disease activity were assessed using Independent Samples T-test for normally distributed data and Mann–Whitney U test for non-normally distributed data. For dichotomous variables, Chi-Square test or Fisher’s exact test was used. Within subgroups of specific TNFi, Spearman’s correlation coefficient was used to analyse correlations of serum trough levels with disease activity as well as BMI. To further explore this association, logistic regression was used with therapeutic and below therapeutic drug levels as dependent variable. Multivariable logistic regression was performed to correct the relation between therapeutic drug levels and disease activity for potential confounders. Each potential confounder was individually added to the multivariable model. The threshold for statistical significance was set at *p* < 0.05 with a confidence interval of 95%.

## Results

### Patient characteristics

One hundred eight axSpA patients were approached for a TNFi serum trough level measurement, of which 67 (62%) had a serum trough level measurement taken within two months from their GLAS visit. Of the 67 patients with a serum trough level measurement, 34 (51%) were treated with adalimumab, 21 (31%) with etanercept, 6 (9%) with infliximab, 4 (6%) with golimumab and 2 (3%) with certolizumab. Further analyses were only performed for patients on adalimumab and etanercept, since number of patients on infliximab, golimumab and certolizumab were too small.

Characteristics of the 55 patients on adalimumab or etanercept with a serum trough level measurement within two months from their GLAS visit (median time 5 days, IQR 0–12) were compared with 36 patients on adalimumab or etanercept without this measurement. The percentage of male patients was significantly higher in the group without a measurement. Furthermore, patients without a measurement had a significantly longer TNFi treatment duration and significantly more often a lower dosage of the TNFi. No differences were seen in disease activity or in diagnosis of AS or non-radiographic axSpA (Supplementary Table 1).

Mean age of the 55 included patients on adalimumab or etanercept was 45 years (SD ± 12), 26 (47%) patients were male, mean symptom duration was 21 years (SD ± 12), and 45 (82%) patients were human leucocyte antigen (HLA)-B27 positive. 46 (84%) patients had a diagnosis of AS and 9 (16%) of non-radiographic axSpA. Median treatment duration of their current TNFi was 41 months (range 2–126, 41 (75%) patients with treatment duration of ≥ 1 year). Median BMI was 26.4 (IQR 23.9–32.5) and median ASDAS was 2.0 (IQR 1.5–3.0) (Table 1).

**Table 1:** Combined and separate characteristics of 55 axSpA patients on adalimumab (*n* = 34) or etanercept (*n* = 21) with TNFi serum trough level measurements

	***Adalimumab and etanercept (n* = *55)***	***Adalimumab (n* = *34)***	***Etanercept (n* = *21)***
*Demographics*			
Age (years)	45 ± 12	45 ± 13	46 ± 12
Sex (male)	26 (47)	14 (41)	12 (57)
BMI (kg/m^2^)	26.4 (23.9–32.5)	27.7 (24.6–33.7)	24.3 (22.2–29.7)*
BMI (kg/m^2^)			
< 25	21 (38)	9 (27)	12 (57)*
25–30	15 (27)	10 (29)	5 (24)
≥ 30	19 (35)	15 (44)	4 (19)
*Disease status*			
Diagnosis of AS	46 (84)	25 (74)	21 (100)**
Duration of symptoms (years)	21 ± 12	21 ± 14	20 ± 8
HLA-B27 positive	45 (82)	27 (79)	18 (86)
History of EAM			
IBD	5 (9)	5 (15)	0 (0)
Psoriasis	7 (13)	5 (15)	2 (10)
Uveitis	12 (22)	8 (24)	4 (19)
ASDAS CRP	2.0 (1.5–3.0)	2.3 (1.7–3.1)	1.6 (1.3–2.0)**
ASDAS ≥ 2.1	25 (46)	21 (64)	4 (19)**
BASDAI (0–10)	3.2 (2.0–5.5)	4.4 (2.8–6.2)	2.6 (1.3–3.5)*
BASDAI ≥ 4.0	21 (39)	17 (52)	4 (19)*
CRP (mg/L)	3.0 (2.0–6.5)	3.9 (2.0–6.1)	2.0 (2.0–6.6)
CRP ≥ 5.0	23 (42)	15 (44)	8 (38)
*Therapy*			
Current NSAID use	18 (33)	12 (36)	6 (29)
Current DMARD use	4 (7)	2 (6)	2 (10)
Previous TNFi	12 (22)	11 (32)	1 (5)*
Treatment duration current TNFi (months)	41 (10–68)	27 (7–57)	60 (18–80)*
Treatment duration since first TNFi (months)	49 (14–87)	41 (14–91)	60 (18–90)
TNFi dosage			
Standard dosage	46 (84)	29 (85)	17 (81)
Higher dosage	2 (4)	2 (6)	0 (0)
Lower dosage	7 (13)	3 (9)	4 (19)
Serum trough level (μg/ml)	NA	5.2 (3.7–8.0)	1.6 (1.1–2.4)

Values are mean ± SD, median (IQR) or n (%)

*Abbreviations: axSpA* axial spondyloarthritis, *TNFi* Tumour necrosis factor (TNF)-α inhibitor, *BMI* Body mass index, *AS* Ankylosing spondylitis, *HLA* Human leukocyte antigen, *EAM* Extra-articular manifestations, *IBD* Inflammatory bowel disease, *ASDAS* AS Disease Activity Score, *CRP* C-reactive protein, *BASDAI* Bath AS Disease Activity Index, *NSAID* Non-steroidal anti-inflammatory drug, *DMARD* Disease-modifying anti-rheumatic drug

^*^Significant difference (*p* < 0.05) compared to patients on adalimumab

^**^Significant difference (*p* < 0.01) compared to patients on adalimumab

Patients on adalimumab showed significantly higher BMI and disease activity, measured with ASDAS and BASDAI compared to etanercept. Furthermore, patients on adalimumab had more often a diagnosis of non-radiographic axSpA, had a shorter treatment duration with their current TNFi and were treated more often with a previous TNFi.

### Adalimumab and etanercept serum trough levels in relation to patient characteristics

Of the 34 patients on adalimumab, median serum trough level was 5.2 (IQR 3.7–8.0) μg/ml. Of the 21 patients on etanercept, median serum trough level was 1.6 (IQR 1.1–2.4) μg/ml (Table 1). Using the definitions of Sanquin, 26 of 55 (47%) patients had a therapeutic serum trough level: 56% of patients on adalimumab and only 33% of patients on etanercept. Of all 29 patients on adalimumab or etanercept with below therapeutic serum trough levels, 7 (24%) patients had a lower dosage than standard, compared to none of the patients with therapeutic serum trough levels (*p* = 0.01). 4 of 15 (27%) patients on adalimumab with below therapeutic levels had presence of anti-drug antibodies, with intermediate titers ranging from 13–22 AU/ml, of which all patients had a standard dosage of their TNFi. 21 (72%) of the patients with below therapeutic serum trough levels had a BMI of ≥ 25 kg/m^2^. In comparison, 13 (50%) of the patients with therapeutic serum trough levels had a BMI of ≥ 25 kg/m^2^ (*p* = 0.09). Of the 21 patients with high BMI there were 5 patients who also had a lower dosage than standard.

**Fig. 1:**
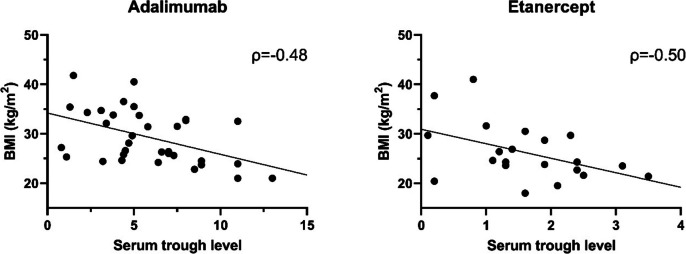
Scatterplot of BMI and serum trough level of adalimumab (**A**) and etanercept (**B**)

Median BMI was higher, although not statistically significant, in the 29 patients with below therapeutic adalimumab or etanercept serum trough levels compared to the 26 patients with therapeutic serum trough levels (28.1, IQR 24.6–34.1 vs. 25.1, 22.8–31.8, *p* = 0.07). Furthermore, BMI had a significant moderate, negative correlation with adalimumab and etanercept serum trough levels (adalimumab: ρ = -0.48, *p* = 0.004, etanercept: ρ = -0.50, *p* = 0.021) (Fig. 1).

### Adalimumab and etanercept serum trough levels and disease activity

No significant differences in disease activity were found between patients with therapeutic or below therapeutic drug levels: median ASDAS was 2.2 (1.6–3.0) vs. 1.9 (1.5–3.1), *p* = 0.53, median BASDAI 3.2 (2.8–5.6) vs. 2.8 (1.9–5.4), *p* = 0.43, median CRP 3.9 (IQR 2.0–6.8) vs. 2.7 (IQR 2.0–6.0), *p* = 0.39.

Furthermore, serum trough levels of adalimumab and etanercept also showed no significant correlation with ASDAS, BASDAI and CRP (Table 2). Similarly, stratifying ASDAS, BASDAI and CRP for ‘active’ and ‘inactive’ disease cut-off values, no significant differences were found in serum trough levels of adalimumab and etanercept (Table 3).

**Table 2:** Correlations of disease activity assessments with serum trough level of adalimumab and etanercept

*Adalimumab*	*Correlation coefficient (ρ)*	*P-value*
ASDAS CRP	-0.16	0.39
BASDAI	-0.14	0.43
CRP	0.002	0.99
*Etanercept*	*Correlation coefficient (ρ)*	*P-value*
ASDAS CRP	-0.29	0.20
BASDAI	-0.17	0.47
CRP	-0.23	0.31

*Abbreviations:* see Table 1

**Table 3:** Median serum trough level of adalimumab and etanercept in groups divided by inactive and active disease or normal BMI and overweight/obesity

	***Serum trough level (µg/ml)***		
	*ASDAS inactive disease (*< *2.1)*	*ASDAS active disease (*≥ *2.1)*	*P-value*
Adalimumab	6.7 (4.0–10.5)	5.0 (3.3–7.8)	0.43
Etanercept	1.4 (1.1–2.4)	1.9 (0.5–2.3)	0.96
	*BASDAI inactive disease (*< *4.0)*	*BASDAI active disease (*≥ *4.0)*	
Adalimumab	7.0 (4.0–8.9)	4.9 (3.3–7.1)	0.23
Etanercept	1.6 (1.2–2.4)	0.9 (0.1–2.1)	0.24
	*CRP* < *5 mg/L*	*CRP* ≥ *5 mg/L*	
Adalimumab	5.3 (4.3–8.0)	5.0 (1.5–8.0)	0.56
Etanercept	1.6 (1.2–2.5)	1.4 (0.4–2.3)	0.37
	*Normal BMI (*< *25 kg/m*^*2*^*)*	*Overweight/obesity (*≥ *25 kg/m*^*2*^*)*	
Adalimumab	8.9 (5.4–11.0)	4.9 (3.3–7.0)	0.012
Etanercept	2.0 (1.3–2.5)	1.2 (0.5–1.8)	0.042

Values are median (IQR)

*Abbreviations:* see Table 1

### Adalimumab and etanercept serum trough levels, disease activity and BMI

Univariable logistic regression showed no significant associations between therapeutic drug levels (yes/no) and disease activity according to ASDAS (OR: 1.19, 95% CI 0.68–2.10), BASDAI (OR: 1.05, 0.83–1.32) or CRP (OR 1.02, 0.97–1.08). Also after correcting for sex, age, BMI, HLA-B27 status, symptom duration, diagnosis of AS or non-radiographic axSpA, current NSAID use, current duration of TNFi therapy or dosage of TNFi, there was no association found (data not shown). However, BMI was identified as confounder on the relationship between therapeutic drug levels and ASDAS (with BMI in model: OR ASDAS 1.44, 95% CI 0.77–2.69).

BMI was significantly correlated with ASDAS (ρ = 0.30, *p* = 0.027) and BASDAI (ρ = 0.31, *p* = 0.022), but not with CRP (ρ = 0.18, *p* = 0.19). Patients with active disease according to ASDAS (≥ 2.1) had higher BMI than patients with inactive disease (median BMI 29.7 vs. 24.6, *p* = 0.015). Similar results were found for high disease activity according to BASDAI (data not shown). Patients with obesity (BMI ≥ 30 kg/m^2^) had higher disease activity than patients with a BMI < 30 kg/m^2^, although not significant (ASDAS: 2.4 vs. 1.8, *p* = 0.07, BASDAI: 4.6 vs. 3.1, *p* = 0.053).

## Discussion

In this cross-sectional observational study of axSpA patients, mainly on long-term treatment with TNFi (median treatment duration of 41 months), BMI was found to be significantly correlated with adalimumab and etanercept serum trough levels. In addition, BMI was significantly higher in patients with active disease. Furthermore, BMI was identified as confounder on the relationship between therapeutic drug levels and disease activity.

We found that 47% of patients had therapeutic serum trough levels, based on Sanquin definitions [14]. Furthermore, 24% of these patients with below therapeutic levels had a lower TNFi dosage than the standard regimen, whereas all patients with therapeutic serum trough levels had normal or higher dosages. Among patients receiving adalimumab with below therapeutic levels, 27% had anti-drug antibodies present, a known factor linked to lower drug levels [22]. For etanercept, it is not possible to measure anti-drug antibodies. Furthermore, higher BMI was significantly associated with below therapeutic serum trough levels, both for adalimumab and etanercept. There were more patients with a BMI of ≥ 25 kg/m^2^ in the group with below therapeutic drug levels compared to those with therapeutic levels, with a trend towards significance.

Given the higher prevalence of obesity in axSpA patients compared to the general population [23], it is important to address the relationship of BMI with low TNFi serum trough levels. Several factors may contribute to low TNFi levels in patients with high BMI. A study in inflammatory bowel disease (IBD) patients aimed at developing a population pharmacokinetic model of adalimumab, demonstrated that higher BMI was associated with increased clearance of adalimumab [24]. Furthermore, obesity may impact the volume of distribution for TNFi [25]. Two cross-sectional studies, one in 273 SpA patients treated with TNFi and one in 57 AS patients treated with adalimumab, also found that high BMI was associated with low serum trough levels [8, 26]. Moreover, a study in 180 axSpA patients on infliximab or adalimumab found that patients with a BMI < 25 kg/m^2^ had more often therapeutic TNFi serum trough levels. Furthermore, they found that use of concomitant conventional synthetic (cs) disease modifying anti-rheumatic drugs (DMARDs) was associated with clinical response in patients with BMI ≥ 25 kg/m^2^ [9]. In our study, concomitant csDMARD use could not be analysed since only 4 patients used this. In axSpA patients it is not common practice to combine TNFi therapy with csDMARDs, unlike patients with peripheral SpA or RA [23, 24].

We not only found that BMI was correlated with serum trough levels, but BMI was also the only confounding factor amongst other clinical variables on the relationship between serum trough levels and ASDAS. Furthermore, BMI was significantly correlated with disease activity measured with ASDAS and BASDAI and patients with active disease according to ASDAS and BASDAI showed higher BMI compared to patients with inactive disease. These results are confirmed by other studies in axSpA patients. Previous studies showed that obesity was associated with higher ASDAS and BASDAI scores, which was most prominent for BASDAI [27]. A study in 624 axSpA patients who recently started with TNFi, showed that obese patients achieved ASAS40 response less frequently compared to patients with a BMI < 25 kg/m^2^ [28]. Similar findings have been reported in other auto-immune conditions such as RA, psoriasis or IBD, where higher BMI has been associated with worse response to TNFi therapy [29, 30]. It could be hypothesized that low drug levels in patients with high BMI may contribute to reduced TNFi response and higher disease activity scores. However, it is worth noting that adipose tissue in obese patients is recognized to be immunologically active, by, for example, secreting pro-inflammatory proteins like TNF [31], which offers an additional potential explanation for the decreased response to TNFi. Because BMI is associated with lower TNFi serum trough levels and higher disease activity, it may be considered to increase the TNFi dosage in this patient group. Another approach might be weight management, as a randomised controlled trial showed that a successful hypocaloric diet in psoriatic arthritis patients resulted in reaching response to TNFi more often compared to patients without weight loss interventions [32]. Another reason to address weight in these patients is that a higher BMI has been associated with more structural damage (e.g. the presence of syndesmophytes) and worse quality of life in axSpA patients [23, 33].

We did not find significant associations between disease activity and TNFi serum trough levels, despite associations of BMI with both disease activity and TNFi levels. Possibly, the group of patients was too small to find an association between disease activity and TNFi levels. The lack of this association may also be related to many patients having a low TNFi serum trough level, especially in the etanercept group, leading to little variation in these drug levels. This is in contrast to other studies, which demonstrated that low TNFi serum trough levels were associated with a lower treatment response and higher disease activity. However, these studies were mostly performed in patients who recently started TNFi therapy [4, 5, 7, 11]. On the other hand, there are also some studies showing no associations between disease activity and TNFi serum trough levels [10, 12].

In our study, relatively more women were included in the group with the TNFi serum trough level measurement, than in the group that did not have this measurement. A possible explanation could be that women have a higher likelihood of responding to research invitations [34]. Furthermore, patients without the serum trough level measurement had a longer treatment duration. Possibly, these patients were more satisfied with their TNFi and therefore less inclined to respond to the invitation for a TNFi serum trough level measurement. However, despite these differences, disease activity and other clinical characteristics were similar across the two groups.

Patients on etanercept experienced lower disease activity according to ASDAS and BASDAI, had a longer treatment duration, lower BMI, and etanercept was more often the first TNFi compared to adalimumab. In our centre it is common practice to start TNFi treatment with etanercept, since etanercept has been registered earlier than adalimumab for AS. If etanercept is not effective or if there are side effects, patients are switched to a different TNFi, which is most often adalimumab. However, the association between BMI, serum trough levels and disease activity occurred both in patients on etanercept and adalimumab.

The main limitation of this study was that the number of patients was too small to perform robust subgroup analyses. Another limitation of this study was that we did not have data on medication compliance. However, all patients receive instructions on the administration of TNFi and at each visit patients are asked for the last date of TNFi administration. Furthermore, the cutoff values for a therapeutic and below-therapeutic range of adalimumab and etanercept were based on reference values of Sanquin, which was mainly based on research in RA and not in axSpA, and especially for etanercept it was difficult to obtain a clear therapeutic window. Because conclusive data regarding cutoff values and specificity and sensitivity is lacking, we mainly focused on TNFi serum trough levels as continuous data in this study [5, 11–13]. A strength of our study is that we studied patients from daily clinical practice of which most patients were on long-term TNFi treatment, whereas previous research regarding TNFi serum trough levels mainly focused on patients who recently started with TNFi therapy.

## Conclusions

To conclude, higher BMI was significantly associated with lower adalimumab and etanercept serum trough levels and higher disease activity assessed with ASDAS and BASDAI. Although adalimumab and etanercept serum trough levels were not associated with disease activity in axSpA patients on long-term treatment, it may be worthwhile to consider increasing the TNFi dosage before switching biological treatment in overweight or obese axSpA patients with low TNFi serum trough levels and high disease activity.

### Supplementary Information

**Additional file 1:** **Supplementary Table 1.** Baseline characteristics of 55 axSpA patients with a random adalimumab or etanercept serum trough level measurement compared to 36 patients on adalimumab or etanercept without measurement.

## Data Availability

Currently, we have no plans to share additional data beyond what is shared in the article.

## Abbreviations

AS: Ankylosing spondylitis
ASDAS: Ankylosing Spondylitis Disease Activity Score
axSpA: Axial spondyloarthritis
BASDAI: Bath Ankylosing Spondylitis Disease Activity Index
BMI: Body mass index
CRP: C-reactive protein
csDMARDs: Conventional synthetic disease modifying anti-rheumatic drugs
ELISA: Enzyme-linked immunosorbent assay
EULAR: European Alliance of Associations for Rheumatology
GLAS: Groningen Leeuwarden Axial Spondyloarthritis
HLA: Human leucocyte antigen
IBD: Inflammatory bowel disease
IQR: Interquartile range
MCL: Medical Centre Leeuwarden
NSAIDs: Non-steroidal anti-inflammatory drugs
OR: Odds ratio
RA: Rheumatoid arthritis
SD: Standard deviation
SPSS: Statistical Package for the Social Sciences
TNFi: Tumour necrosis factor alpha inhibitor
UMCG: University Medical Centre Groningen
