# Supplementary material for: High BMI is associated with lower TNF-α inhibitor serum trough levels and higher disease activity in patients with axial spondyloarthritis
Source: Arthritis Res Ther. 2023 Oct 17;25:202. doi: 10.1186/s13075-023-03187-4 (PMC10580642; doi:10.1186/s13075-023-03187-4)
Supplement: Supplementary file 1 — Additional file 1: Supplementary Table 1. Baseline characteristics of 55 axSpA patients with a random adalimumab or etanercept serum trough level measurement compared to 36 patients on adalimumab or etanercept without measurement. [file 13075_2023_3187_MOESM1_ESM.pdf]

SUPPLEMENTARY MATERIAL

Supplementary Table 1: Baseline characteristics of 55 axSpA patients with a random adalimumab or etanercept serum trough level measurement compared to 36 patients on adalimumab or etanercept without measurement

Demographics	*All patients with measurement (n=55)*	*Without measurement (n=36)*
Age (years)	45 ± 12	47 ± 12
Sex (male)	26 (47)	30 (83)*
BMI (kg/m^2)	26.4 (23.9-32.5)	26.0 (23.9-29.8)
Disease status		
Diagnosis of AS	46 (84)	30 (83)
Duration of symptoms (years)	21 ± 12	24 ± 12
HLA-B27 positive	45 (82)	27 (75)
History of EAM		
IBD	5 (9)	8 (22)
Psoriasis	7 (13)	3 (8)
Uveitis	12 (22)	14 (39)
ASDAS CRP	2.0 (1.5-3.0)	1.9 (1.4-2.7)
BASDAI (0-10)	3.2 (2.0-5.5)	2.3 (1.2-5.1)
CRP (mg/L)	3.0 (2.0-6.5)	3.6 (2.0-6.4)
Therapy		
Current NSAID use	18 (33)	15 (43)
Current DMARD use	4 (7)	2 (5)
Previous TNFi	12 (22)	12 (33)
Treatment duration current TNFi (months)	41 (10-68)	81 (34-108)*

Treatment duration since first TNFi (months)	49 (14-87)	84 (37-108)**
Dosage of TNFi		
Standard dosage	46 (84)	23 (64)**
Higher dosage	2 (4)	1 (3)
Lower dosage	7 (13)	12 (33)**
Specific TNFi used		
Adalimumab	34 (62)	18 (50)
Etanercept	21 (38)	18 (50)

Values are mean ± SD, median (IQR) or n (%)

*Significant difference ($p<0.005$) compared to all patients with a serum trough level measurement

**Significant difference ($p<0.05$) compared to all patients with a serum trough level measurement

Abbreviations: axSpA: axial spondyloarthritis; TNFi: tumour necrosis factor (TNF)-α inhibitor; BMI: body mass index; AS: ankylosing spondylitis; HLA: human leukocyte antigen; EAM: extra-articular manifestations; IBD: inflammatory bowel disease; ASDAS: AS Disease Activity Score; CRP: C-reactive protein; BASDAI: Bath AS Disease Activity Index; NSAID: non-steroidal anti-inflammatory drug; DMARD: disease-modifying anti-rheumatic drug
